# Follow-up after neonatal heart disease repair: watch out for chronic kidney disease and hypertension!

**DOI:** 10.1007/s00467-020-04621-4

**Published:** 2020-06-04

**Authors:** Louis Huynh, Sara Rodriguez-Lopez, Kelly Benisty, Adrian Dancea, Daniel Garros, Erin Hessey, Ari Joffe, Rachel Joffe, Andrew Mackie, Ana Palijan, Alex Paun, Michael Pizzi, Michael Zappitelli, Catherine Morgan

**Affiliations:** 1 Faculty of Health Sciences, School of Medicine, Queen’s University, Kingston, ON Canada; 2 Department of Pediatrics, Division of Nephrology, University of Alberta, Stollery Children’s Hospital, Room 4-555, 11405-87 Avenue, Edmonton, AB T6G 1C9 Canada; 3 Faculty of Medicine, McGill University, Montreal, QC Canada; 4 Department of Pediatrics, Division of Cardiology, Montreal Children’s Hospital, McGill University Health Centre, Montreal, QC Canada; 5 Department of Pediatrics, Division of Pediatric Critical Care, University of Alberta, Edmonton, AB Canada; 6 Faculty of Medicine, University of Alberta, Edmonton, AB Canada; 7 Department of Pediatrics, Division of Cardiology, University of Alberta, Edmonton, AB Canada; 8 McGill University Health Centre Research Institute, McGill University Health Centre, Montreal, QC Canada; 9 Department of Pediatrics, Division of Nephrology, Peter Gilgan Centre for Research and Learning, Toronto Hospital for Sick Children, 686 Bay Street, Room 6th floor 9708, Toronto, ON M5G 0A4 Canada

**Keywords:** Congenital heart disease, Outcomes, Chronic kidney disease, Hypertension, Acute kidney injury, Neonate

## Abstract

**Background:**

With advances in care, neonates undergoing cardiac repairs are surviving more frequently. Our objectives were to 1) estimate the prevalence of chronic kidney disease (CKD) and hypertension 6 years after neonatal congenital heart surgery and 2) determine if cardiac surgery-associated acute kidney injury (CS-AKI) is associated with these outcomes.

**Methods:**

Two-center prospective, longitudinal single-visit cohort study including children with congenital heart disease surgery as neonates between January 2005 and December 2012. CKD (estimated glomerular filtration rate < 90 mL/min/1.73m^2^ or albumin/creatinine ≥3 mg/mmol) and hypertension (systolic or diastolic blood pressure ≥ 95th percentile for age, sex, and height) prevalence 6 years after surgery was estimated. The association of CS-AKI (Kidney Disease: Improving Global Outcomes definition) with CKD and hypertension was determined using multiple regression.

**Results:**

Fifty-eight children with median follow-up of 6 years were evaluated. CS-AKI occurred in 58%. CKD and hypertension prevalence were 17% and 30%, respectively; an additional 15% were classified as having elevated blood pressure. CS-AKI was not associated with CKD or hypertension. Classification as cyanotic postoperatively was the only independent predictor of CKD. Postoperative days in hospital predicted hypertension at follow-up.

**Conclusions:**

The prevalence of CKD and hypertension is high in children having neonatal congenital heart surgery. This is important; early identification of CKD and hypertension can improve outcomes. These children should be systematically followed for the evolution of these negative outcomes. CS-AKI defined by current standards may not be a useful clinical tool to decide who needs follow-up and who does not.

## Introduction

Epidemiological studies have shown the prevalence of cardiac surgery-associated acute kidney injury (CS-AKI) to be as high as 50–60% in neonates [1, 2]. While much is known about the short-term outcomes of these patients, including prolonged ventilation, prolonged intensive care admission, and longer hospital stay [1, 3], there is less known about the long-term renal outcomes such as chronic kidney disease (CKD) and hypertension. Two recent studies of pediatric patients showed no association of CS-AKI (measured by changes in serum creatinine) with long-term renal outcomes [4, 5]. However, these studies did not include neonates, who have a unique renal physiology compared with the general pediatric population and perhaps have different critical care risks [6].

With advances in care, neonates undergoing complex cardiac repairs are surviving more frequently, resulting in a markedly increasing number of adults with congenital heart disease [7, 8]. Given the lack of knowledge of long-term renal function in neonates, there is a need to further elucidate the risk of CKD and the role of neonatal CS-AKI in this vulnerable patient population. Chronic kidney disease causes significant personal and economic healthcare burden and is associated with worse long-term outcome, quality of life, and well-being in the general population [9]. It is a condition where early identification and intervention to mitigate modifiable risks can delay progression. Currently, there are no evidence-based guidelines for the follow-up of neonates after congenital heart repair relevant to renal health.

We hypothesized that CKD and hypertension are common in children who undergo neonatal congenital heart surgery and that CS-AKI increases long-term risk for CKD and hypertension. Our objectives were to 1) estimate the prevalence of CKD and hypertension 5–7 years after neonatal cardiac surgery and 2) determine if CS-AKI is a risk factor for later CKD and hypertension.

## Materials and methods

### Study cohort and recruitment

This is a two-center, longitudinal, cohort study of neonates who underwent neonatal congenital heart surgery at the Montreal Children’s Hospital, Montreal, Canada, and the Stollery Children’s Hospital, Edmonton, Canada, between January 2005 and December 2012. Exclusion criteria were preoperative known kidney disease, already recruited into a longitudinal study, unwillingness to return to the study center for assessments, or lived too far away (> 3.5-h drive) from the center for home study visits. Institutional Research Ethics Board approval was obtained before initiating this study. Written informed consent was provided by parents/legal guardians prior to initiating study activities with participants.

### Study population source

Patients were identified (via hospital databases), reviewed for eligibility, and mailed invitations to contact us for study information. Responders were contacted, re-reviewed for eligibility, and invited to participate. In addition, children attending cardiology clinics in both Edmonton and Montreal fitting eligibility criteria were invited to participate, and children previously consenting to future contact during prior studies were contacted by telephone.

### Study procedure

A single standardized study visit was performed 5 to 7 years post-cardiac surgery at the clinic research center or the patient’s home. Study staff were blind to past AKI status. Blood (minimum 1.5 mL) and urine (minimum 5 mL) samples were collected. Three measures of height (by either SECA 217 stadiometer, SECA; standardized supine measurement; or measuring tape if limited mobility) and weight (UC-321 PL Precision Health scale, A&D Medical; barefoot; bulky clothing removed) were taken, and the average was calculated. Height and weight, age and gender-specific percentiles, and z-scores were calculated using Centers for Disease Control and Prevention growth charts [10]. Three automated blood pressure (BP) measurements were performed (Omron HEM-711AC, Omron Healthcare, Inc.; regularly calibrated) in a quiet setting (efforts to reduce anxiety; before blood work), seated, using size-appropriate cuffs on the right arm (unless contraindicated). The average of the two lowest blood pressure measurements was used to calculate the age, gender, and height-specific blood pressure percentile. Blood pressure percentiles were calculated according to the 2017 American Academy of Pediatrics blood pressure guideline [11]. Data was collected on the use of anti-hypertensive medications.

### Index cardiac surgery data

Relevant clinical data from the index cardiac surgery admission were collected retrospectively by chart review. Preoperative variables collected included sex, gestational age (weeks), age (days), weight (kg) and length (cm) at surgery, baseline serum creatinine (SCr, defined as the last creatinine drawn before surgery), ventilated at the time of surgery, and use of preoperative extracorporeal membrane oxygenation (ECMO). Complexity of cardiac surgery was categorized using the risk adjustment for congenital heart surgery-1 (RACHS-1) [12], the consensus-based scoring system used during the operative course of the included neonates, with higher scores indicating more complex surgeries. We also documented the more objective STAT (The Society of Thoracic Surgeons-European Association for Cardio-Thoracic Surgery) mortality category [13] for further evaluation of the cohort. Postoperative variables included daily SCr values, cyanotic postoperatively (defined by postoperative hypoxemia (low partial pressure of oxygen as measured on arterial blood gas) and compatible heart lesion), duration of invasive mechanical ventilation, PICU stay greater than 1 week, duration of hospital stay, dialysis treatment, and postoperative nephrotoxic medication use within the first week after surgery (categorized as yes or no and included NSAIDs, aminoglycosides, acyclovir/ganciclovir, amphotericin, or vancomycin).

### Study visit laboratory data

Specimens were kept on ice, centrifuged at 2000 RPM for 15 min at 21 °C, separated into aliquots, and stored at − 80 °C until analysis at the Montreal Children’s Hospital laboratory (blinded to clinical data). Blood was measured for SCr (enzymatic, isotope-dilution mass spectrometry-traceable). Urine was measured for albumin (nephelometry, Prospec II, Siemens) and creatinine (modified Jaffe assay).

### Primary exposure

Our primary exposure was CS-AKI, defined based on the creatinine criteria of Kidney Disease: Improving Global Outcomes (KDIGO) definition (≥ 50% rise or ≥ 26.5-μmol/L increase in postoperative SCr from baseline). SCr within the first 7 days postoperatively was used to define CS-AKI. Baseline SCr value was defined as the last SCr value collected before index cardiac surgery. We also evaluated moderate/severe CS-AKI (SCr at least doubling or dialysis).

### Outcomes

Our primary outcomes were CKD and hypertension. Hypertension was defined as a systolic or diastolic blood pressure ≥ 95th percentile for age, sex, and height [11], or a history of hypertension currently being treated with medication. For descriptive purposes, elevated blood pressure was defined as a systolic or diastolic blood pressure ≥ 90th percentile for age, sex, and height [11]. Chronic kidney disease was defined as an estimated glomerular filtration rate (eGFR) < 90 mL/min/1.73 m^2^ or urine albumin/creatinine (ACR) ≥ 3 mg/mmol (stage 2 CKD) [14]. Estimated GFR was calculated using the SCr and height-based bedside CKiD equation [15].

### Statistical analysis

Statistical analysis was performed using the STATA (14.0) statistical software (College Station, TX). Statistical significance was set at *p* value ≤ 0.05. Descriptive analyses are reported as frequency and proportion for categorical variables, median with interquartile range (IQR) for continuous variables with non-normal distribution, and mean with standard deviation (SD) for continuous variables with normal distribution. Associations of CS-AKI and other variables with the outcomes were evaluated using linear regression or the Mann-Whitney *U* tests (for continuous variables and depending on distribution), and *χ*^2^ or Fisher’s exact test (for categorical variables). Purposeful selection was the regression strategy planned, with the following variables considered for the prediction model: sex, gestational age (term vs not), age at surgery, weight at surgery, baseline eGFR, ventilated at surgery, cyanotic postoperatively, duration of postoperative invasive mechanical ventilation, duration of postoperative ICU and hospital stays, and postoperative nephrotoxic medication use. We planned to evaluate RACHS-1 score for univariable relationship with outcomes; however, we planned to exclude it from multivariable modeling as it is a consensus-based scoring system used to categorize surgical complexity and predict outcome in the setting of limited primary data collection; it encompasses data likely to be included in other variables. In addition, for evaluation of hypertension outcome, we built 2 separate models, one including those with arch abnormalities or coarctation and one without. Variables with *p* < 0.2 in univariate analysis were to be included as potential predictive variables in multiple regression modeling. Given the small sample size, we predicted the ability to use multiple logistic regression to evaluate independent association of CS-AKI, and the outcomes may be limited. We estimated that we may only be able to control for the effects of up to three other variables (approximately 5 to 10 outcomes per variable). A priori, we decided that if greater than 3 variables (other than CS-AKI) were associated with the outcome, we would use stepwise, backward elimination logistic regression analysis (with *p* value for exclusion from the model > 0.15), retaining the CS-AKI variable. Separate logistic regression models were evaluated for each of the outcomes. Assumptions of regression were tested.

Given the challenges of using creatinine-based definitions of AKI in neonates and the potential for misclassification of our primary exposure measure, we assessed the relationship between preoperative SCr, eGFR, age at surgery, and age at baseline SCr measurement (each separately), and a diagnosis of CS-AKI. Associations were evaluated using Student’s *t* tests or the Mann-Whitney *U* tests, as appropriate.

## Results

### Patient characteristics

**Fig. 1:**
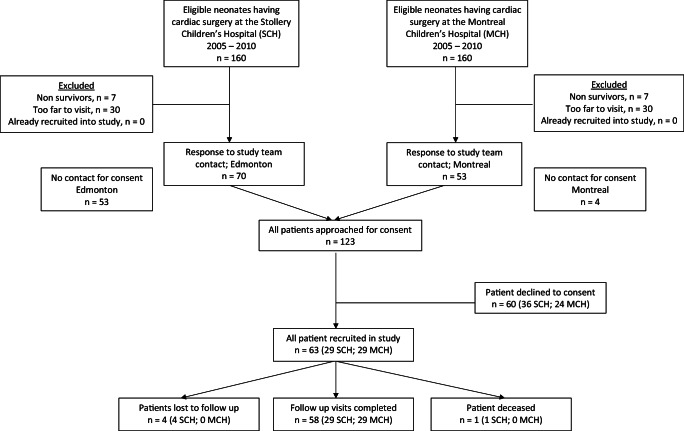
Study flow

Fifty-eight neonates were enrolled in the study (Fig. 1). Response rate of patients contacted by study site is shown in Fig. 1. Consent rate of patients who responded was 51% (Fig. 1). Enrolled patients did not differ in sex, age at cardiac repair, or CS-AKI prevalence from a previously prospectively recruited cohort studied by our group (evaluated > 90% of neonatal biventricular repairs done over a 7-year period in Edmonton) [1]. Thirty-seven (64%) were male, and 47 (81%) were born at term. Cardiac surgery occurred at a median (IQR) age of 10 (10) days of life with a mean (SD) weight of 3.2 (0.8) kg. Seventy-two percent were ventilated at the time of surgery, but none of them needed ECMO pre- or postoperatively. The most common RACHS-1 category was category 3, accounting for approximately half of the neonates (54%) with 5%, 21%, 10%, and 10% of neonates in the remaining categories 1, 2, 4, and 6 respectively. There was no RACHS-1 category 5. This distribution is similar to the general pediatric cardiac surgery population, with category 5 being uncommon, and categories 2 and 3 being the most common [12]. The most common STAT category was category 4, accounting for 62% of the neonates, with 11%, 15%, and 13% of neonates in the categories 1, 2, and 5 respectively. There were no neonates in category 3.

AKI status was available in all patients. Fifty-seven percent (33/58) of the cohort had a history of CS-AKI; 33% of those had stage 1 AKI, 15% had stage 2, and 9% had stage 3. Only 2 required postoperative dialysis. Ten of the 33 neonates (30%) who were classified as having CS-AKI did not recover their baseline SCr before discharge. Eighty-three percent of neonates (47/58) had a baseline preoperative creatinine drawn within 48 h of cardiac surgery, with almost all of these being drawn within 24 h. Creatinine used to determine CS-AKI status was drawn more than 7 days preoperatively in 4 children; 3 were classified as having AKI and 1 was not. Another 6 neonates had the SCr drawn more than 48 h before surgery but less than 7 days. Of those, 4 were classified as having CS-AKI and 2 were not. In these, 2 classified as non-AKI; the SCr was drawn the first and second days of life. Mean (SD) of age at baseline SCr measurement was 9.3 (7.2) days. Neonates who were classified as having CS-AKI had a lower baseline SCr (regression coefficient − 17.4, 95% CI − 29.1 to − 5.2, *p* = 0.006) and a higher baseline eGFR (regression coefficient 14.3, 95% CI 3.2 to 25.3, *p* = 0.02) compared with those who were classified as not having CS-AKI. Age at cardiac surgery and age at baseline SCr measurement were not associated with a diagnosis of CS-AKI.

The mean (SD) number of years between index cardiac surgery and follow-up visit was 6.3 (0.9), which did not differ between CS-AKI groups (*p* = 0.52). At the follow-up study visit, weight for age percentile and height for age percentile distributions in the study population were not different than the general pediatric population (*p* = 0.70 and 0.23 for weight and height respectively). Mean (SD) weight for age percentile was 50 (28). Only 4 children had a weight for age less than the 10th percentile and none had a weight < 5th percentile. Mean (SD) height for age percentile was 55 (29). Only 2 children had a height for age less than the 10th percentile, with both being less than the 5th percentile and both also having a weight for age less than the 10th percentile. There was no statistically significant difference in height, weight, or age in patients who had CS-AKI compared with those that did not.

### Renal outcome characteristics

Out of fifty-eight patients, 53 had blood samples to estimate glomerular filtration rate, 58 had urine samples collected, and 57 had blood pressure measurements performed during the follow-up visit. The prevalence of CKD in our study cohort was 17% (9/53). Mean (SD) eGFR was 123 (20.5) mL/min/1.73m^2^. No patient had an eGFR by SCr < 60 mL/min/1.73m^2^. Mean (SD) follow-up time for those with CKD and those without CKD was 6.6 (0.9) years and 6.2 (0.9) years, respectively (*p* = 0.11). Overall, the point prevalence of microalbuminuria in our study sample was 12% (7/58), and the median (IQR) albumin to creatinine ratio was 1.43 (1.3) mg/mmol. Of the seven patients who had microalbuminuria, only one had an ACR greater than 20 mg/mmol. Thirty percent (17/57) were classified as hypertensive, with an additional 16% (9/57) being classified as having elevated blood pressure. If those with arch abnormalities or coarctation were excluded, 27% (13/48) were classified as hypertensive.

#### Predictors of renal outcomes

**Fig. 2:**
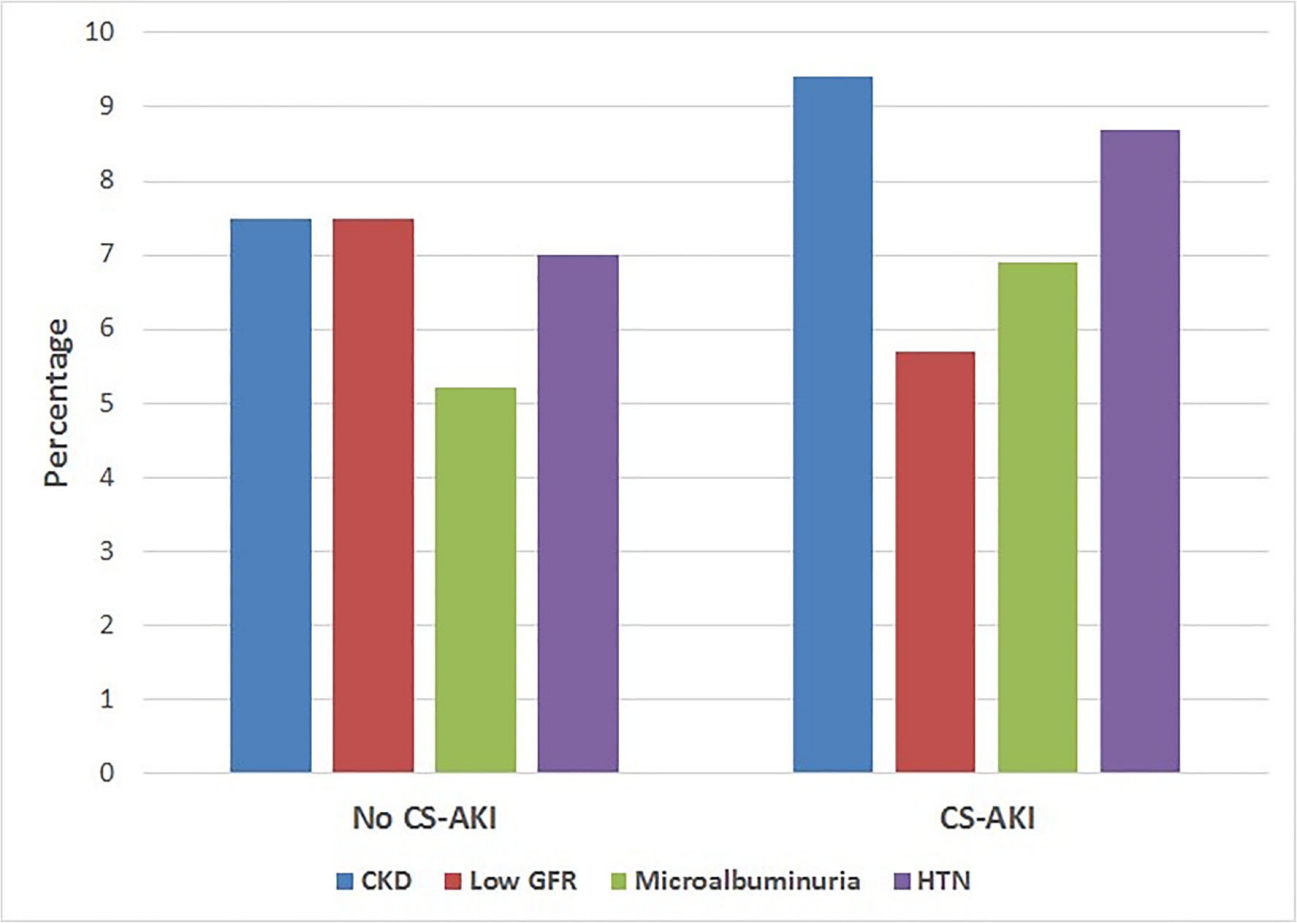
Prevalence of 6-year kidney outcomes by cardiac surgery-associated acute kidney injury. CS-AKI, cardiac surgery-associated acute kidney injury; CKD, chronic kidney disease; HTN, hypertension; Low GFR, estimated glomerular filtration rate less than 90 mL/min/1.73m^2^

Patient and surgical characteristics of children with and without CKD are shown in Table 1, and long-term renal outcomes following cardiac surgery by CS-AKI status are shown in Fig. 2. Multiple-variable analysis demonstrated that classification as cyanotic postoperatively was the only independent predictor of CKD (adjusted OR 18, 95% CI 3.1 to 105.4, *p* = 0.001). CS-AKI was not statistically significantly associated with CKD, even in moderate and severe episodes of CS-AKI. Multiple-variable analysis demonstrated that postoperative days in hospital were the only independent predictor of hypertension (HTN) at follow-up (adjusted OR 1.08, 95% CI 1.02 to 1.15, *p* = 0.007). CS-AKI was also not statistically significantly associated with hypertension even in moderate and severe episodes of CS-AKI. Given the potential association between coarctation or arch abnormalities with longer-term hypertension, we repeated the analysis for predictors of hypertension excluding these children (*n* = 9), and when these patients were removed from the analysis of predictors of HTN, postoperative hospital days remained the only significant predictor.

**Table 1:** Characteristics by CKD and HTN status and univariate comparisons

Characteristic	CKD (*n* = 9)	No CKD (*n* = 44)	*p* value	HTN (*n* = 17)	No HTN (*n* = 40)	*p* value
Gestational age ≥ 37 weeks, *n* (%)	7 (78)	35 (79)	0.90	13 (76)	33 (82)	0.59
Weight at surgery, kg, mean (SD)	3.2 (1)	3.2 (1)	0.96	3.3 (0.6)	3.2 (1)	0.89
Age at surgery, days, median (IQR)	7 (6)	10 (11)	0.17	10 (9)	9.5 (12)	0.79
Preoperative mechanical ventilation, *n* (%)	7 (78)	31 (70)	0.65	14 (82)	28 (70)	0.33
Preoperative eGFR, mL/min/1.73 m^2^, median (IQR)	51 (41)	46 (27)	0.55	46 (27)	45 (34)	0.91
Sex, male, *n* (%)	4 (44)	30 (68)	0.17	13 (76)	24 (60)	0.23
Surgical category, *n* (%)			0.04			0.82
RACHS-1 category 1	1 (11)	2 (4)		1 (6)	2 (5)	
RACHS-1 category 2	2 (22)	10 (23)		3 (18)	9 (23)	
RACHS-1 category 3	2 (22)	25 (57)		8 (47)	22 (55)	
RACHS-1 category 4	1 (11)	5 (11)		2 (11)	4 (10)	
RACHS-1 category 5	0 (0)	0 (0)		0 (0)	0 (0)	
RACHS-1 category 6	3 (34)	2 (4)		3 (18)	3 (7)	
Cyanotic postoperatively, *n* (%)	7 (78)	7 (16)	< 0.001	5 (30)	10 (25)	0.63
Postoperative nephrotoxic medication^a^ use in ICU, *n* (%)	3 (33)	18 (41)	0.67	6 (35)	16 (40)	0.73
Postoperative mechanical ventilation, days, median (IQR)	7 (5)	6 (5)	0.57	7 (6)	6 (5)	0.75
Postoperative ICU stay >1 week, *n* (%)	2 (22)	25 (56)	0.06	11 (69)	20 (50)	0.31
Length of postoperative hospital stay, days, median (IQR)	17 (16)	22 (19)	0.34	31 (21)	26 (14)	0.01

^a^Nephrotoxic medication: NSAIDs, aminoglycosides, acyclovir/ganciclovir, amphotericin, vancomycin

**Fig. 3:**
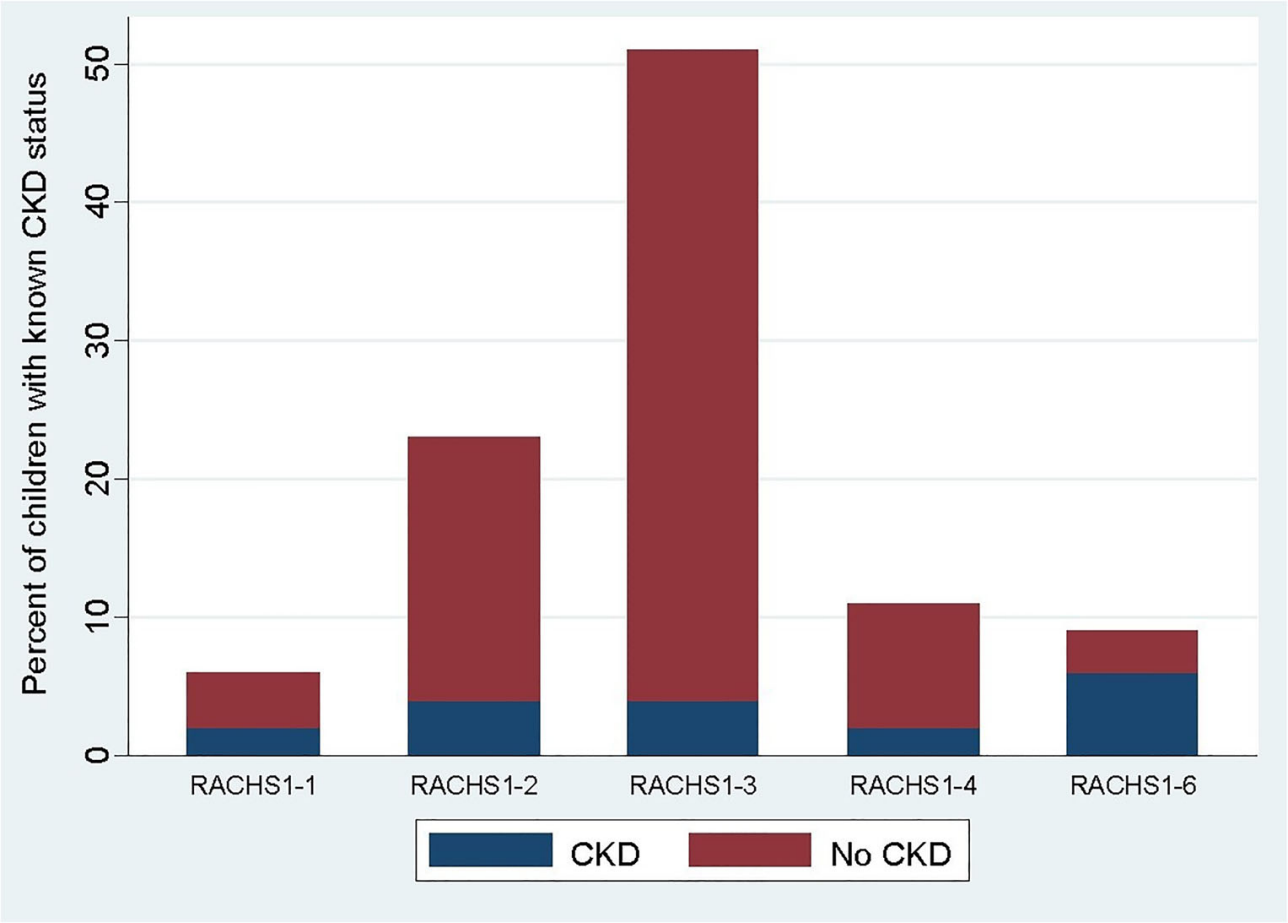
Distribution of children with CKD by RACHS-1 category. RACHS, risk adjustment for congenital heart surgery; CKD, chronic kidney disease

RACHS-1 category for type of surgical repair was associated with CKD (*p* = 0.04). The distribution of children with CKD by RACHS-1 category is shown in Fig. 3. Congenital heart repairs in 33% of children with CKD in the study cohort were RACHS-1 category 6. Category 6 also had the highest prevalence of CKD, with 60% of children developing CKD. The association of STAT category with CKD was also significant (*p* = 0.025), with the highest prevalence in STAT category 5 (66%). No children in STAT 1 were classified as CKD, and 14% of both STAT 2 and STAT 4 had CKD. There was no association identified between either RACHS-1 category or STAT category and hypertension at follow-up.

## Discussion

This is the first multicenter study evaluating both the childhood prevalence of and potential risk factors for CKD and hypertension long term after neonatal congenital heart repair. The prevalence of both CKD and HTN was higher in our study cohort compared with the general pediatric population. In Canadian children, the reported CKD prevalence is 2.1% compared with 17% in our study [16]. Alarmingly, 30% of our study population had hypertension, compared with a prevalence of 0.8% in otherwise healthy Canadian children, measured at a single study visit with similar methodology to our study [17]; an additional 15% were classified as having elevated blood pressure in our study. Preterm neonates are thought to have higher risk of elevated blood pressure; however, the majority of children in the current study had been born at term and we found no association between gestation and blood pressure. Consideration was also given to the possibility that hypertension in this cohort could be driven by persistent abnormalities unrelated to the kidneys, following repair of some types of defects, particularly coarctation and arch abnormalities. However, we did not find any significant association between these specific defects, and the outcome of hypertension in this cohort and the prevalence of hypertension remained very high (27%) even after these children were excluded.

A significant relationship between these long-term outcomes and the development of CS-AKI was not found in our study, even after moderate and severe episodes of CS-AKI. Higher prevalence of CKD and hypertension may represent a consequence of renal damage occurring in the context of cardiac surgery, critical illness, and immature kidneys that are not detected by the current standard definition of AKI. Non-creatinine-based renal injury biomarkers may be a useful tool in further evaluating this. It is also possible that we misclassified some of the neonates regarding their AKI status, leading to non-significant results between CS-AKI and long-term renal outcomes. Neonates who were classified as having CS-AKI had a significantly higher baseline eGFR and lower baseline SCr compared with those who were classified as not having CS-AKI. Baseline Scr may have reflected maternal SCr, and the SCr peak after surgery may have not been enough to classify them as having CS-AKI, even if true injury occurred. We did however observe that age of the neonate at surgery and age at baseline SCr measurement were not associated with a diagnosis of CS-AKI. AKI, defined by the current standard of using serum creatinine, may not be a useful indicator for dictating follow-up in neonates, given the complexities of this biomarker in this population. In this study, we did not assess for multiple AKI events over early childhood as a risk factor for CKD. It may be that cumulative kidney injury is more important than a single isolated event; children with congenital heart disease often have multiple hospitalizations as well as repeat surgical intervention, particularly those with cyanotic heart disease.

Classification as cyanotic postoperatively was associated with higher prevalence of CKD. In cyanotic congenital heart disease, chronic hypoxemia leads to increase blood viscosity, which has been shown experimentally to influence endothelial shear stress, glomerular arteriolar resistance, hydraulic pressure within the glomerulus, and filtration fraction [18], which can lead to chronic nephropathological changes including glomerulosclerosis [19, 20]. Local renal tissue hypoxia may have a role in the initiation and progression of CKD in this population. In the renal tubular epithelium and glomerular mesangial cells, hypoxia is mitogenic and fibrogenic [21, 22]; experimental evidence suggests that renal tubular structure is abnormal and interstitial fibrosis is present after a relatively short period of tissue hypoxia [23]. Complexity of surgery/risk of mortality scores was not analyzed in multiple-variable modeling, but in the analysis of its univariate relationship with the outcomes, they did predict CKD. Children with RACHS-1 category 6 or STAT category 5 repairs had a higher incidence of CKD; all of these children were also classified as cyanotic, which may explain the observed association.

Hypertension was significantly associated with total length of stay in hospital after cardiac surgery. Total length of stay is likely a surrogate marker of overall illness condition, with sicker children staying longer. Children who stay longer may be at higher risk of multiple interventions, with the potential to impact the kidney as well as recurrent exposure to nephrotoxins, which could lead to elevated blood pressure in the long term.

This study has a number of limitations. The sample size was small. Although it was large enough to identify meaningful differences in outcomes between the study cohort and the reference populations, it was likely not large enough to detect small differences in subgroups of patients. The small sample size also limited confounder adjustment. Albuminuria was diagnosed on a single spot urine, with 44% of the cohort with CKD being diagnosed as such by albuminuria alone. Although current guidelines recommend a spot urine for definition of albuminuria, a first morning urine in the study setting may give a more accurate assessment of this outcome, given the potential postural effects on protein excretion. There are very few studies on the prevalence of albuminuria from random spot urines in the child population, particularly in the ages included in our study. However, given the age at follow-up of the current cohort, the incidence of orthostatic proteinuria is likely low [20]. There are challenges in using creatinine-based estimation equations in determining CKD status in a variety of different pediatric populations, and we do not know the best estimation equation for determining GFR in a cohort of children with a history of congenital heart disease. We identified the commonly used SCr and height-based bedside CKiD equation to be the most appropriate, but we do not know this to be empirically true. Given the possibility of white coat hypertension, hypertension prevalence may have been overestimated. Even if half of the children had white coat hypertension, the prevalence of blood pressure abnormalities would still have been very high. Outcome measures were determined at a single point in time, which may not represent the chronic trajectory of these children. Lastly, sampling bias places some limitations on the external generalizability of the findings of our study; only 23% of the total eligible population was studied. We did demonstrate that baseline demographics were likely similar to the general eligible population, and RACHS score distribution was similar to previous large cohorts evaluated; however, there may have been selection bias towards patients with more critical illness, as these are the subjects more likely to follow up.

## Conclusion

The prevalence of CKD and hypertension is high in children having had congenital heart repair as neonates. Although high prevalence of CKD has been shown in adults with congenital heart disease, this study demonstrates that there is evidence of negative renal consequences long before adulthood. This is important as hypertension and albuminuria may be modifiable contributing factors in CKD progression. This study provides evidence for healthcare providers to systematically follow these children over time for the evolution of these negative outcomes. Although the current study has limitations, CS-AKI defined by current standards did not predict CKD in this cohort. This needs further evaluation in a larger study to determine if CS-AKI can be a useful clinical tool in deciding who needs follow-up and who does not. The advent of new biomarkers may further elucidate this problem, allowing for a better understanding of the pathophysiology and possible prevention of hypertension and CKD in this vulnerable population.

## Data Availability

The datasets used and/or analyzed during the current study are available from the corresponding author on reasonable request.
